# Effect of lidocaine pumped through hepatic artery to relieve pain of hepatic artery infusion chemotherapy

**DOI:** 10.3389/fsurg.2024.1378307

**Published:** 2024-08-07

**Authors:** Renjie Zhang, Yiling Liao, Xiaoya Yang, Hengyu Tian, Shenfeng Wu, Qingteng Zeng, Qinghua He, Ruikun Zhang, Chunshan Wei, Jialin Liu

**Affiliations:** ^1^ Department of General Surgery, Shenzhen Traditional Chinese Medicine Hospital, The Fourth Clinical Medical College of Guangzhou University of Chinese Medicine, Shenzhen, Guangdong, China; ^2^ The Fourth Clinical Medical College of Guangzhou University of Chinese Medicine, Shenzhen, Guangdong, China; ^3^ Department of Hepatology, Shenzhen Traditional Chinese Medicine Hospital, The Fourth Clinical Medical College of Guangzhou University of Chinese Medicine, Shenzhen, Guangdong, China

**Keywords:** hepatocellular carcinoma, hepatic arterial infusion chemotherapy, lidocaine, pain, visual analogue score, comfort score

## Abstract

**Background:**

This study aims to explore the analgesic effect of lidocaine administered through the hepatic artery during hepatic artery infusion chemotherapy (HAIC) for hepatocellular carcinoma (HCC).

**Methods:**

A total of 45 HCC patients were randomly divided into a study group and a control group. Both groups received oxaliplatin (OXA) based FOLFOX protocol via electronic infusion pump. The study group was continuously infused with 100 mg of lidocaine during HAIC, while 5% glucose solution was infused in the same way as described above. Changes in vital signs, visual analogue score (VAS) and general comfort score (GCQ scale) were recorded before surgery (Time point 0), at the end of infusion (Time point 01), 1 h after HAIC (Time point 02), 3 h after HAIC (Time point 03) and 6 h after HAIC (Time point 04).

**Results:**

At each point of time from Time point 0 through Time point 04, the differences in MAP, RR and SPO_2_ between the two groups were not statistically significant (*P* > 0.05). At each point of time from Time point 01 through Time point 04, the mean VAS scores in the study group were smaller and GCQ scores were higher than those in the control group, and the differences were both statistically significant (*P* < 0.05).

**Conclusions:**

Lidocaine infusion through the hepatic artery during HAIC effectively reduces intraoperative and postoperative pain and improves patient satisfaction with pain management, making it a valuable technique for clinical practice.

## Introduction

Hepatocellular carcinoma (HCC) is a primary liver cancer with a high mortality rate, significantly contributing to the global cancer burden (1–5). Most patients are diagnosed in the middle to advanced stages, missing the optimal window for surgery and subsequently requiring chemotherapy (6, 7). Hepatic artery infusion chemotherapy (HAIC) is an effective treatment for unresectable HCC (8–10). Recently, the FOLFOX combination strategy, which includes folinic acid (FnA), 5-fluorouracil (5-FU), and oxaliplatin (OXA), has become prominent in HAIC, significantly enhancing the tumor response rate and patient survival rate for middle and advanced liver cancer (11–14). However, many HCC patients undergoing HAIC experience substantial pain due to arterial spasms caused by the continuous infusion of chemotherapy drugs (15–17). This often leads to the suspension of treatment, negatively affecting therapeutic outcomes. To enhance the quality of life and the effectiveness of HAIC treatment, various interventions, such as non-steroidal anti-inflammatory drugs, opioids, intramuscular anti-spasmolytics, and intra-arterial lidocaine injections, are commonly used in clinical practice (16, 18), despite their limited analgesic efficacy and the potential need for repetitive use.

Lidocaine is the predominant anesthetic used in HAIC, administered through arterial or intravenous routes to provide analgesia (16, 19). However, challenges such as dilution by hepatic artery and the liver microenzyme-mediated degradation of lidocaine to the intermediate metabolite monoethyl glycine xylene pose limitations, resulting in a relatively short-lived analgesic effect (16, 20). To improve patient comfort during surgery and ensure the smooth progression of HAIC treatment, prolonging the analgesic effect is a key clinical objective.

Our study reveals that hepatic artery infusion of lidocaine significantly reduces intraoperative and postoperative pain in HCC patients undergoing HAIC, providing a satisfactory analgesic effect. The details of our findings are presented in the following report.

## Materials and methods

### Patients

A total of 45 HCC patients, who underwent treatment at Shenzhen Traditional Chinese Medicine Hospital between January 2021 and August 2023, were randomly selected as the subjects for this study. Approval for this study was obtained from the Ethics Committee of Shenzhen Traditional Chinese Medicine Hospital, and all patients participated voluntarily, were duly informed, and signed an informed consent form.

### Inclusion and exclusion criteria

Inclusion criteria were as follows: (1) Diagnosis based on liver cancer criteria. (2) Age ≥18 years. (3) Indications for HAIC without drug allergies. (4) Child-Pugh score grade A to B. (5) Obtained written consent.

Exclusion criteria were as follows: (1) Severe functional impairment of the heart, lungs, kidneys, or other organs. (2) History of mental illness, mental disorders, clouding of consciousness, or communication disorders. (3) Allergies or intolerances. (4) Voluntary withdrawal from study participation. (5) Prolonged history of opioid use.

### HAIC method

**Figure 1:**
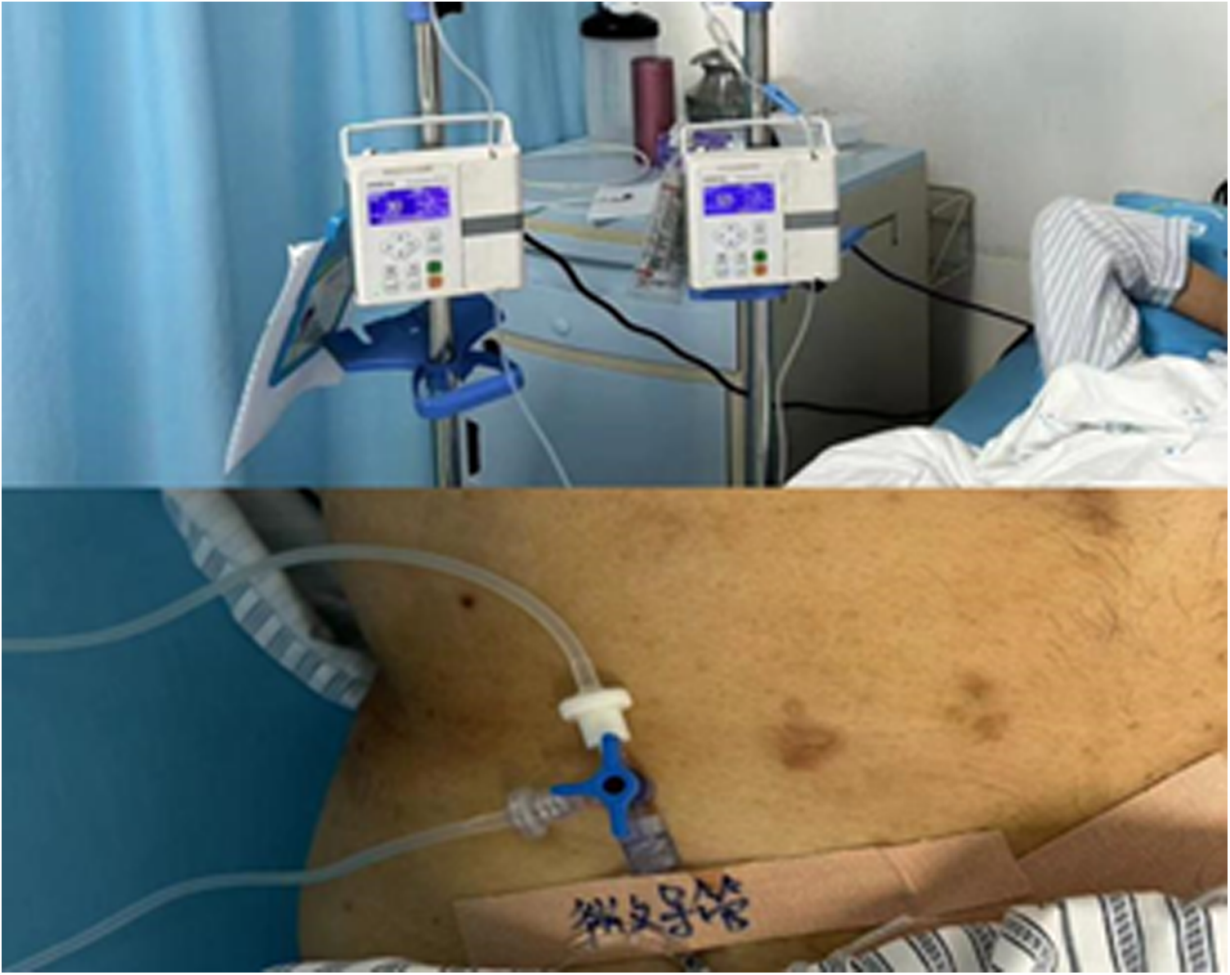
HAIC patients with lidocaine pumped through the hepatic artery.

The Seldinger method was used for femoral artery intubation, employing a 5F-catheter to identify the blood supply artery of the tumor. After catheter fixation, HCC patients in both groups received continuous arterial infusion of chemotherapy drugs while in bed. During infusion, care was taken to avoid bending and exerting force on the catheter to prevent displacement. The study group received a continuous infusion of 100 mg lidocaine during HAIC, with 5% glucose solution infused in a similar manner as described above (Figure 1). The control group received a single intravenous injection of 50 mg lidocaine during HAIC if the pain was intolerable. Supplemental morphine was administered if additional analgesia was needed.

### Chemotherapeutics

The chemotherapeutic regimen used in this study is FOLFOX, a combination of folinic acid (FnA), 5-fluorouracil (5-Fu), and oxaliplatin (OXA). The specific protocol includes OXA at a dose of 85 mg/m^2^ administered via a hepatic arterial pump for 2–3 h, FnA at a dose of 400 mg/m^2^ administered via a hepatic arterial pump for 1–2 h, and 5-Fu at a dose of 400 mg/m^2^ initially through arterial nolus, followed by continuous arterial infusion of 2,400 mg/m^2^ for 23 h.

### Observational index

The changes of vital signs (including mean arterial blood pressure (MAP), heart rate (HR), respiratory rate (RR), SpO_2_) were recorded before surgery (Time point 0), at the end of perfusion (Time point 01), 1 h after HAIC (Time point 02), 3 h after HAIC (Time point 03), and 6 h after HAIC (Time point 04); Visual analgesia scores at each time point (VAS score: 0: no pain;1–3: mild pain; 4–6 points: moderate pain, 7–9 severe pain;10: unbearable pain); and high scores indicate severe pain. Comfort score at each time point (GCQ scale: using 1–4 scale scoring method, that is, 1 means “strongly disagree”, 2 means “disagree”, 3 means “agree”, 4 means “strongly agree”), the higher the score, the higher the comfort.

### Statistical analysis

Data analysis was conducted using SPSS 26.0 statistical software. Measurement data were expressed as mean and standard deviation and analyzed using the Student's *t*-test. The χ^2^ test was employed for group comparisons, with a significance level set at *P* < 0.05.

## Results

### General conditions

**Figure 2:**
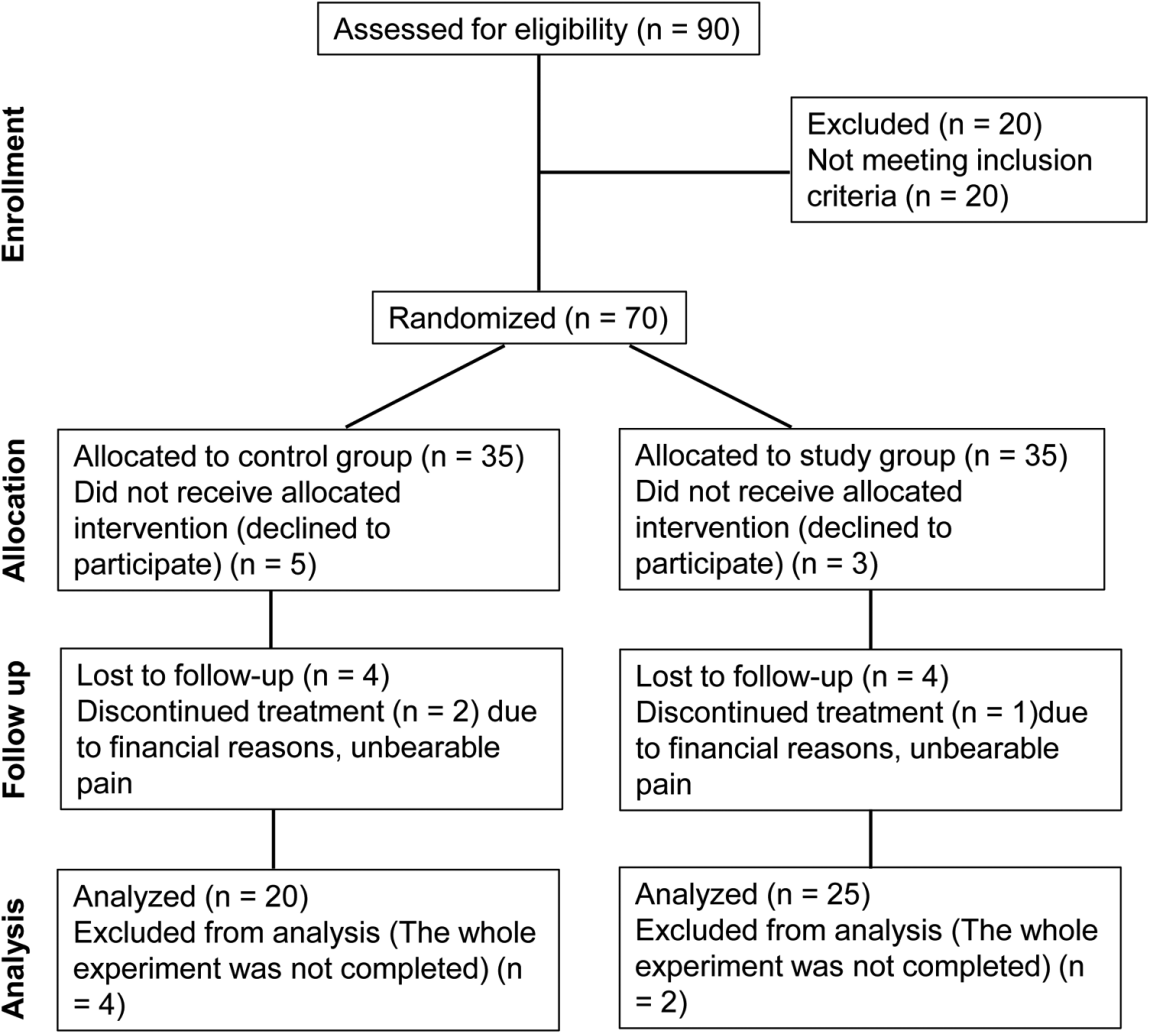
CONSORT diagram of the study.

The study design was summarized using a flow chart based on the CONSORT diagram (Figure 2). Patients were divided into a study group and a control group. The control group comprised 14 males and 6 females, with a mean age of 52.8 ± 6.3 years old and a mean hepatic artery diameter of 3.59 ± 0.71 mm. The study group consisted of 18 males and 7 females, with a mean age of 52.6 ± 7.1 years old and a mean hepatic artery diameter of 3.68 ± 0.68 mm. Patients in both groups successfully completed the experiment, and there were no statistically significant differences in the baseline clinical characteristics between the two groups (*P* > 0.05) (Table 1).

**Table 1:** Baseline characteristics of patients.

	Control group (*n* = 20)	Study group (*n* = 25)	*P*-value
Age (years)			
≤60	14 (70%)	18 (71%)	>0.05
>60	6 (30%)	7 (28%)	
Gender			
Male	12 (60%)	16 (64%)	>0.05
Female	8 (40%)	9 (36%)	
Tumor number			
≤3	12 (60%)	13 (62%)	>0.05
>3	8 (40%)	12 (48%)	
AFP (ng/ml)			
≥400	11 (55%)	15 (60%)	>0.05
<400	9 (45%)	10 (40%)	
ALB (g/L)	40.28 ± 4.36	39.01 ± 4.22	>0.05
TB (μmol/L)	21.17 ± 8.77	22.75 ± 11.12	>0.05
PLT (×10^9^)	188.49 ± 75.92	192.73 ± 94.58	>0.05
PT (S)	12.22 ± 1.31	12.57 ± 1.30	>0.05
Cr (μmol/L)	74.20 ± 15.36	73.65 ± 16.02	>0.05
Hepatic artery diameter (mm)	3.68 ± 0.68	3.59 ± 0.71	>0.05
Hepatitis			
HBV	16 (80%)	22 (88%)	>0.05
HCV	2 (10%)	1 (4%)	
No hepatitis	2 (10%)	2 (8)	
Family history			
Yes	13 (65%)	18 (72%)	>0.05
No	7 (35%)	7 (28%)	
Drinking history			
Yes	7 (35%)	9 (36%)	>0.05
No	13 (65%)	16 (64%)	
Child-Pugh			
A	16 (80%)	19 (76%)	>0.05
B	4 (20%)	6 (24%)	
BCLC stage			
B	17 (85%)	20 (80%)	>0.05
C	3 (15%)	5 (20%)	

AFP, alpha fetoprotein; ALB, albumin; BCLC stage, Barcelona clinic liver cancer stage; Cr, creatinine; HBV, hepatitis B virus; HCV, hepatitis C virus; PLT, platelet; TB, total bilirubin.

### Vital signs

There were no significant differences in MPA, RR, HT, and SpO_2_ between the two groups at Time point 0, Time point 01, Time point 02, Time point 03, and Time point 04 (*P* > 0.05) (Table 2).

**Table 2:** Vital signs of HCC patients in two groups at different time points.

	Control group	Study group	*P*-value
MAP (mmHg)			
Time point 0	102.6 ± 8.85	105.6 ± 10.63	>0.05
Time point 01	103 ± 9.03	104.6 ± 8.87	>0.05
Time point 02	104.5 ± 10.17	105.8 ± 9.62	>0.05
Time point 03	102.6 ± 8.69	103.1 ± 7.83	>0.05
Time point 04	103.6 ± 9.40	104.4 ± 8.95	>0.05
HR (beat/min)			
Time point 0	68.55 ± 5.02	68.7 ± 4.59	>0.05
Time point 01	67.0 ± 3.48	67.2 ± 3.70	>0.05
Time point 02	66.6 ± 4.15	66.9 ± 3.92	>0.05
Time point 03	67.6 ± 8.69	66.8 ± 5.23	>0.05
Time point 04	66.9 ± 4.59	67.3 ± 4.34	>0.05
RR (Breath/min)			
Time point 0	16.5 ± 1.43	16.5 ± 1.45	>0.05
Time point 01	16.0 ± 1.00	15.9 ± 0.97	>0.05
Time point 02	16.1 ± 1.07	16.2 ± 1.18	>0.05
Time point 03	16.7 ± 1.45	16.7 ± 1.46	>0.05
Time point 04	16.3 ± 1.02	16.3 ± 1.14	>0.05
SpO_2_/%			
Time point 0	97.2 ± 1.04	97.0 ± 1.19	>0.05
Time point 01	97.2 ± 1.04	96.7 ± 1.02	>0.05
Time point 02	96.6 ± 0.88	96.6 ± 1.04	>0.05
Time point 03	96.6 ± 0.94	96.4 ± 0.99	>0.05
Time point 04	96.8 ± 1.06	96.8 ± 1.13	>0.05

Time point 0, preoperative; Time point 01, at the end of perfusion; Time point 02, 1 h after HAIC; Time point 03, 3 h after HAIC; Time point 04, 6 h after HAIC. HR, heart rate; MAP, mean arterial pressure; RR, respiratory rate.

### VAS scores

There was no statistical significance in the comparison of VAS at Time point 0 between the study group and the control group (*P* > 0.05). However, a statistically significant difference in VAS scores was observed from Time point 01 to Time point 04 between the study group and the control group (*P* < 0.05) (Table 3). Moreover, the scores in the study group after treatment were consistently lower than those before treatment. Notably, at the end of HAIC perfusion, four patients in the control group experienced severe pain, necessitating repeated use of morphine for pain relief. In contrast, in the study group, two patients reported severe pain at the end of HAIC perfusion, and their pain was alleviated by increasing the dose of lidocaine. No cases in the study group required supplementary morphine analgesia post-surgery.

**Table 3:** Comparison of VAS scores of HCC patients treated with HAIC between the two groups.

VAS scores	Control group	Study group	*P*-value
Time point 0	1.55 ± 0.51	1.68 ± 0.63	>0.05
Time point 01	1.85 ± 1.46	0.92 ± 1.15	<0.05
Time point 02	2.10 ± 0.97	0.84 ± 0.99	<0.05
Time point 03	2.35 ± 0.59	0.88 ± 0.87	<0.05
Time point 04	2.45 ± 0.51	0.84 ± 0.47	<0.05

Time point 0, preoperative; Time point 01, at the end of perfusion; Time point 02, 1 h after HAIC; Time point 03, 3 h after HAIC; Time point 04, 6 h after HAIC. VAS, visual analogue score.

### GCQ scores

There was no statistical significance in the GCQ score at Time point 0 between the study group and the control group (*P* > 0.05). However, the GCQ scores of the study group exhibited a continuous increase from Time point 01 to Time point 04, and the difference was statistically significant compared to the control group (*P* < 0.05) (Table 4).

**Table 4:** Comparison of GCQ scores in HCC patients treated with HAIC between the two groups.

GCQ scores	Control group	Study group	*P*-value
Time point 0	1.95 ± 0.76	2.08 ± 0.86	>0.05
Time point 01	1.55 ± 0.51	2.84 ± 0.75	<0.05
Time point 02	1.75 ± 0.55	2.96 ± 0.54	<0.05
Time point 03	1.65 ± 0.59	3.08 ± 0.40	<0.05
Time point 04	2.05 ± 0.39	3.24 ± 0.52	<0.05

Time point 0, preoperative; Time point 01, at the end of perfusion; Time point 02, 1 h after HAIC; Time point 03, 3 h after HAIC; Time point 04, 6 h after HAIC; GCQ, general comfort score.

## Discussion

Approximately 90% of the blood supply to liver cancer tissue is derived from the hepatic artery (21). HAIC allows the continuous infusion of high-concentration cytotoxic drugs directly into tumors, maximizing their lethal effect while minimizing the distribution of chemotherapy drugs to other organs (22, 23). This approach produces potent anti-tumor effects, reduces systemic side effects, and significantly improves the overall survival of patients with liver cancer (24). The FOLFOX regimen, based on OXA, is an approved systemic chemotherapy regimen for advanced liver and colorectal cancer, demonstrating survival benefits in patients with advanced cancer (22).

The lethal effect of chemotherapy drugs on tumor cells may induce a local inflammatory response leading to pain. OXA-induced peripheral neuropathy is a common and sometimes treatment-limiting side effect (25). In clinical practice, during HAIC with OXA for HCC patients, severe abdominal pain, nausea, and vomiting resulting from vasospasm and other adverse reactions can occur, leading to treatment discontinuation. Studies have also suggested that the diameter of the hepatic artery contributes to abdominal pain during HAIC, particularly when the catheter is used for drug infusion (16).

In our study, we found no statistically significant factors affecting pain scores, such as OXA preparation and hepatic artery diameter. During HAIC perfusion, a 100 mg lidocaine infusion through the three-way tube showed a noticeable intraoperative analgesic effect, likely due to reduced nociceptive stimulation from chemotherapy drugs and effective inhibition of central or peripheral nerve sensitization.

Lidocaine, a local anesthetic, is known for its fast, potent, and long-lasting effects, as well as its wide safety range. In the arterial system, lidocaine acts as a potent vasodilator, particularly relaxing highly strained vascular smooth muscle (26). Studies have confirmed that injecting 100 mg of lidocaine through the hepatic artery before embolization effectively relieves intraoperative pain (12). The mechanism behind intraarterial lidocaine's analgesic effect may involve local effects from diffusion into the vascular wall and hepatic parenchyma, endovascular surface anesthesia, and direct vasodilation. This effect can be prolonged by blocking tumor blood supply and slowing drug clearance (12, 27). Previous studies have demonstrated that intra-arterial lidocaine injection is an effective method in most patients (96%, 361 of 376) (16). However, direct injection of lidocaine through the hepatic artery has limitations, resulting in reduced analgesic efficacy and shortened duration due to blood scour and dilution. In comparison to the direct intra-arterial injection method, we used an electronic pump to infuse 100 mg lidocaine through the hepatic artery. Compared to the control group, the study group exhibited favorable changes in preoperative, intraoperative, and postoperative vital signs, VAS, and GCQ scores. Results indicated lower VAS scores in the study group at Time point 01–04 compared to the control group, with post-treatment VAS scores lower than pre-treatment scores. No cases in the study group required supplementary morphine for analgesia, while some patients in the control group needed morphine for pain relief. Lidocaine infused through the hepatic artery significantly reduced intraoperative pain and provided prolonged postoperative analgesia. Additionally, patients in the study group reported higher satisfaction and comfort levels than those in the control group (*P* < 0.05), affirming the positive analgesic effects of hepatic artery lidocaine infusion and its suitability for clinical promotion.

Furthermore, our results indicated that increasing the lidocaine dose in patients with low pain tolerance was effective, maintaining stable vital signs without adverse events (20). Nevertheless, the maximum effective and safe dose of lidocaine pumped through the hepatic artery during HAIC operations requires further investigation.

## Conclusions

In conclusion, lidocaine infusion through the hepatic artery during HAIC effectively reduces intraoperative and postoperative pain and improves patient satisfaction with pain management, making it a valuable technique for clinical practice.

## Data Availability

The original contributions presented in the study are included in the article/Supplementary Material, further inquiries can be directed to the corresponding author.
